# Universal neonatal audiological screening: experience of the University Hospital of Pisa

**DOI:** 10.1186/1824-7288-37-16

**Published:** 2011-04-11

**Authors:** Paolo Ghirri, Annalisa Liumbruno, Sara Lunardi, Francesca Forli, Antonio Boldrini, Angelo Baggiani, Stefano Berrettini

**Affiliations:** 1 Mother and Child Department, Neonatology Unit and Section of Neonatal Endocrinology and Dysmorphology, University Hospital of Pisa, Pisa, Italy; 2 Division of ENT, Department of Neuroscience, University of Pisa, Pisa, Italy; 3 Department of Experimental Pathology, Medical Biotechnologies, Infectious Diseases and Epidemiology, University of Pisa, Pisa, Italy

## Abstract

The early identification of pre-lingual deafness is necessary to minimize the consequences of hearing impairment on the future communication skills of a baby. According to the most recent international guidelines the deafness diagnosis must occur before the age of three months and the prosthetic-rehabilitative treatment with a traditional hearing aid should start within the first six months. When a Cochlear implant becomes necessary, the treatment should start between the age of 12 months and 18 months. The only way to diagnose the problem early is the implementation of universal neonatal audiological screening programs. Transient evoked otoacoustic emissions (TEOAE) is the most adequate test because it's accurate, economic and of simple execution. Automatic auditory brainstem response (AABR) is necessary to identify patients with auditory neuropathy but it is also important to reduce the number of false-positives.The 20-30% of infant hearing impairment is represented by progressive or late-onset hearing loss (HL) so it's also necessary to establish an audiological follow up program, especially in infants at risk.

From November 2005 all neonates born in the University hospital of Pisa undergo newborn hearing screening. From 2008 the screening program follows the guidelines for the execution of the audiological screening in Tuscany which have been formulated by our group according to the 2007 JCIH Position Statement and adaptated to our regional reality by a multidisciplinary effort. From November 2005 to April 2009 8113 neonates born in the Neonatal Unit of Santa Chiara Hospital (Pisa) have undergone newborn hearing screening. 7621 neonates (93.9%) without risk factors executed only the TEOAE test. 492 (6.1%) neonates had audiological risk factors and thus underwent TEOAE and AABR. 84 patients (1,04%) failed both TEOAE and AABR tests. 78 of them underwent further investigations. 44 patients resulted falsepositives (the 0,54% of the screened newborns). 34 neonates (4,2 ‰) had a final diagnosis of hearing impairment. 8 patients (0.99 ‰) had unilateral hearing loss (HL). 26 patients (3,2 ‰) had bilateral hearing impairment.

In our screening program the percentage of false-positives was quite low (0.54%) while the incidence of bilateral HL (3.2 ‰) is a little higher than that found in literature reports. In most of our patients premature birth or neonatal suffering represent the main cause of HL.

## Introduction

The identification and the early diagnosis of pre-lingual deafness is necessary to prevent or minimize the serious consequences of hearing impairment on language development and on the future communication skills of a baby [1-7].

According to the most recent international guidelines the deafness diagnosis must occur before the age of three months and the prosthetic-rehabilitative treatment with a traditional hearing aid should start within the first six months. When a Cochlear implant becomes necessary, the treatment should start between the age of 12 months and 18 months [8-14]. Pre-lingual deafness is a silent pathology which often becomes evident only after causing serious consequences on the acquisition and development of language abilities. The only way to early diagnose the problem is the implementation of universal neonatal audiological screening programs [5].

The aim of such programs is to identify hearing impairments present at birth, overall medium and severe (bilateral, >= 40 dB HTL between 0.5 and 4 KHz) [15-21,6,7].

The most important international guidelines suggest the execution of a universal screening program and hence screening tests should be done on all neonates and not only on those presenting increased risk factors [5-7,22], as only about half of the babies suffering from permanent hearing conditions present increased risk factors[22-28].

Actually in Italy neonates without risk factors are tested at around 8 months of age with the use of the *Boel *test. *Boel test *is difficult to execute (it requires specific expertise) and it successfully identifies only less than half of babies with a hearing impairment. That explains why, in the absence of a screening program, the average delay in the diagnosis ranges between 18 months and 24 months. Such delay might cause a decreased effectiveness of the rehabilitation therapy and irreparable consequences for the patient [7,12-14,29,30].

The aim of the universal screening is hence to identify as early as possible the highest number of infants with permanent bilateral hearing impairments [6,7,15,22,31].

Recent screening methodologies (TEOAE and AABR) are completely risk-free and extremely accurate [6,7,32].

When the latest technology is used in conjunction with specific operator training, the accuracy of screening programs can be close to 100% with a specificity of about 97-98%. That means that virtually all neonates with a hearing impairment greater than 40dB will be identified, while about 2-3% of the babies diagnosed will be false positives. The false positives are later re-examined and a large part of them subsequently presents normal hearing function [33-35].

In the last 10 years a new clinical/audiological entity has been defined (but not completely understood): the auditory neuropathy. Auditory neuropathy is characterised by normal otoacoustic emissions and altered auditory brainstem response, due to damage either to the inner hair cells, damage to the acoustic nerve fibres or to the synaptic junction between them and inner hair cells [32,36-38].

Neonates affected by auditory neuropathy can be diagnosed only by executing the ABR test (automatic or clinical) in conjunction with otoacoustic emissions. A TEOAE test in this cases would lead to a falsely negative outcome. For these reasons it is necessary to test neonates with auditory neuropathy risk factors with both TEOAE and AABR tests [7,36].

In the last few years there has been an increased focus on late onset hearing loss. Progressive or late onset deafness can have different causes (genetic predisposition, infections etc.) and represents a relatively large percentage (20-30%) of hearing loss in children, even if reliable statistics are not available internationally [39]. Babies affected with progressive or late onset deafness may not be identified with neonatal hearing screening and might be identified only by a long term paediatric surveillance program.

The Joint Committee on Infant Hearing (JCIH) in the 2007 Position Statement has identified the issue of late onset hearing loss and has defined the risks factors that requires an audiological follow-up during the first years of life [7].

With the Regional Decree n.365 of May 21st 2007, the local regional government of Tuscany (*Regione Toscana*) has made compulsory the execution of the neonatal audiological screening program in all the birth centers of the region.

We have subsequently written the "Guidelines for the execution of the neonatal audiological screening" together with the Department of Audiology of the Careggi Hospital (headed by Prof P Pagnini) and with the Audiology Department of the University of Siena (headed by Prof W Livi). The guidelines were approved by the *Consiglio Sanitario Regionale Toscano *(Tuscan Regional Health Council) in June 2008 and are today a reference for all the hospitals and operators (neonatologists, paediatricians, otorhinolaryngologists, audiologists, audiometrists, paediatric nurses, child neuropsychiatrists, geneticists) executing screening programs in Tuscany. Our guidelines are based on the ones created by the JCIH in 2007, but have been modified by a multidisciplinary effort and adapted to our regional reality. Follows a brief example showing the methods of screening execution in Tuscany.

### Screening execution details in Tuscany

The screenings can be executed in three different types of hospitals which are distinguished by their level. A centre is assigned a level depending on the type of instruments and diagnostic equipment available.

**Level I facilities: **these facilities can only execute TEOAE tests with latest available diagnostic instruments. The test is executed by audiometrists, audiologists, paediatricians, paediatric nurses or otorhinolaryngologists after a specific training.

**Level II facilities: **these facilities have the latest available instruments necessary to execute TEOAE and AABR tests. Also in this case the test is executed by trained audiometrists, audiologists, paediatricians, paediatric nurses or otorhinolaryngologists.

**Level III facilities **(reference hospitals): these hospitals have the latest equipment for the execution of TEOAE, AABR and they are also capable of executing clinical ABR, clinical TEOAE and DPOAE (distortion produced otoacoustic emissions), impedence audiometry and infant audiometric testing.

All these diagnostic tests are executed by personnel with a specific expertise in this field. Level III facilities can perform an early diagnosis, an investigation on the causes of the hearing impairment and the initiation of a prosthetic/rehabilitative treatment. Each facility should at first classify neonates depending on whether they present auditory neuropathy risk factors or no because well babies and neonates at risk should perform different kind of screening test battery.

In all cases the screening must be executed before discharging the neonate from hospital and positive tests might be repeated within two weeks after birth. Each local health authority should nominate a person in charge of the screening procedures in each facility. This person might be a paediatrician, neonatologist, audiologist or an otorhinolaryngologist and he/she should involve the audiometrists and the paediatric nurses in the process. In general is advised that the screening should be performed only by dedicated personnel with a specific training and not by generic health operators. Especially in facilities of Level II and III, the screening should be performed by audiometrists and there should be a close collaboration between the Neonatal Units and the Audiology Units.

The Clinical Physiology Institute of the CNR (National Council for Research) is developing a database that will be used by all regional facilities to file and retrieve neonatal audiological screening data. Such data will then be made available online. This will allow a monitoring on the effectiveness of the screening program (a screening program is considered adequate when it's executed on more than 95% of neonates and it identifies at least 99% of the hearing loss cases with only less than 2-3% of false positives).

All these entities will need to establish the details of the screening execution for the babies born in private clinics, babies born at home, in other Italian regions or other countries. All these babies will need to be screened within a month from birth or within a month from the moment they are assigned to a family paediatrician.

#### 1. Screening in well babies (fig 1)

**Figure 1:**
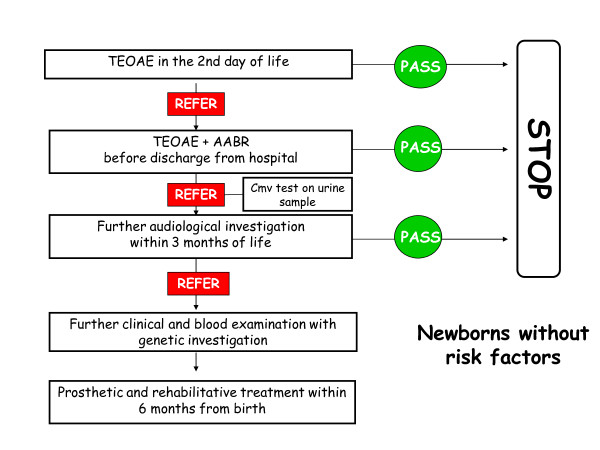
**Graphical algoritm of screening investigation in neonates without audiological risk factors**.

Dedicated personnel (audiometrists, neonatologist, audiologist, otorhinolaryngologist or paediatric nurses) should execute the TEOAE test 24 hours after birth. Tests should be performed in silent rooms while the neonates are asleep or when they are most quiet (for example after feeding). If the test outcome is negative (*pass*) for both ears the audiological screening is considered successfully completed. If instead the test outcome is positive (*refer*) for one or both ears then the TEOAE test should be repeated before the infant is discharged from hospital. If this second test still produces a positive result the infant should undergo a AABR test. Level I facilities should send these neonates to level II or level III facilities where AABR can be performed. AABR should be executed within 30 days from birth. If the complete test (TEOAE+AABR) outcome is positive, infants should undergo further tests in a level III facility, within 90 days from birth, such as clinical ABR, clinical TEOAE etc. Level III facilities should then start the investigation on the causes of the hearing impairment and should initiate a prosthetic/rehabilitative treatment within 6 months from birth.

Congenital infections from Cytomegalovirus (CMV) should be immediately investigated in all infants that result positive at the first level audiological screening (TEOAE), researching CMV DNA in urine.

#### 2. Screening in neonates with auditory neuropathy risk factors (fig 2)

**Figure 2:**
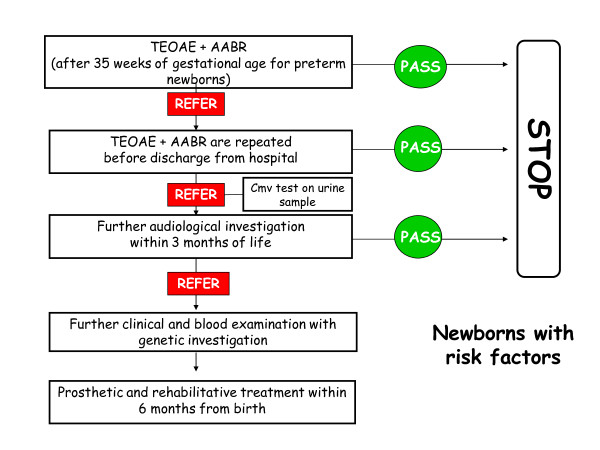
**Graphical algoritm of screening investigation in neonates with audiological risk factors**.

Auditory neuropathy risk factors are:

- neonatal intensive care of more than 5 days or any of the following regardless of length of stay (ECMO, assisted ventilation, exposure to ototoxic medications such as gentamycin and tobramycin or loop diuretics such as furosemide, and hyperbilirubinemia that requires exchange transfusion)

- family history of permanent childhood hearing loss

- family history of neurodegenerative disorders, such as Hunter syndrome, or sensory motor neuropathies, such as Friedreich ataxia and Charcot-Marie-Tooth syndrome.

Neonates with auditory neuropathy risk factors should undergo both automatic TEOAE and AABR test before the discharge from the hospital (or within 30 days from birth if neonates are born in a level I facility).

In premature neonates audiological screening should be executed after 35 weeks of gestational age. If the test produces a positive result it should be repeated before discharge or within two weeks. In all infants where audiological tests produce a definitely positive result a Cytomegalovirus (CMV) infection should be investigated by detection of CMV DNA in urine. If the second test still produces a positive result the infant should be sent to level III ORL Department to perform further tests within 3 months from birth.

Before the infant is discharged from the hospital the person responsible for the screening should write the screening results on the paediatric booklet (this booklet will be given to the infant's family when the neonate is discharged from the hospital), on the screening's results register and on the online database. The person responsible should specify which kind of test (TEOAE, AABR or both) the neonate has undergone and what result (positive or negative) the test has produced. The screening manager should also specify the timing for further audiological investigation depending on neonate's risk factors. Parents should be given adequate information on congenital hearing impairment if their neonate has failed newborn hearing screening. Infants of a few months of age re-admitted to hospital within because of hyperbilirubinemia requiring exsanguino-transfusion or for a sepsis diagnosed with positive culture should repeat TEOAE and AABR test regardless to their neonatal screening result.

### Infantile progressive deafness and late-onset hearing impairment

Progressive and late-onset deafness account for about 20-30% of infantile hearing loss. These patients can develop later hearing impairment even if the neonatal audiological screening was successfully completed. The only way to identify them is to keep monitoring infants with risk factors for progressive or late-onset hearing impairment throughout their childhood regardless to their neonatal audiological screening result. Patients with the following risk factors should undergo at least one audiological evaluation between 24 and 30 months of age:

- neonatal intensive care of more than 5 days or any of the following regardless of length of stay (ECMO, assisted ventilation, exposure to ototoxic medications such as gentamycin and tobramycin or loop diuretics such as furosemide, and hyperbilirubinemia that requires exchange transfusion),

- In-utero infections, such as herpes, rubella, syphilis, and toxoplasmosis

- Craniofacial anomalies, including those that involve the pinna, ear canal, ear tags, ear pits, and temporal bone anomalies

- Physical findings, such as white forelock, that are associated with a syndrome known to include a sensorineural or permanent conductive hearing loss.

Boel test should be performed by the family paediatrician as usual.

Patients with the risk factors listed below should undergo audiological evaluation every 6-12 months till 3 years of age and then every 12 months till 6 years of age.

- In-utero CMV infection

- family history of permanent childhood hearing loss

- Syndromes associated with hearing loss or progressive or late-onset hearing loss, such as neurofibromatosis, osteopetrosis, and Usher syndrome; other frequently identified syndromes include Waardenburg, Alport, Pendred, and Jervell and Lange-Nielson.

- Neurodegenerative disorders, such as Hunter syndrome, or sensory motor neuropathies, such as Friedreich ataxia and Charcot-Marie-Tooth syndrome

- Culture-positive postnatal infections associated with sensorineural hearing loss, including confirmed bacterial and viral (especially herpes viruses and varicella) meningitis

- Head trauma, especially basal skull/temporal bone fracture that requires hospitalization

- Chemotherapy or ototoxic drugs

- Caregivers concern regarding hearing, speech, language, or developmental delay

Boel test should be performed by the family paediatrician as usual.

Family paediatricians should be part of his screening program by investigating auditory function in infants and children with and without risk factors during routine health checks at 3,6,8,12,18 months and at 3 and 6 years.

## Experience of the University Hospital of Pisa

### Patients and methods

From 1993 to the 20th of november 2005 neonates with audiological risk factors have been evaluated with clinical ABR.

From the 20th of November 2005 all neonates born in the University hospital of Pisa (which represents the reference facility of the North-West Tuscany Area) undergo newborn hearing screening. They execute the test (TEOAE or AABR depending on the presence or absence of auditory risk factors) before the discharge from hospital or within 2 weeks from birth. This test, and further investigations in newborns presenting positive test outcome (*refer*) can be performed thanks to the collaboration between the *U.O. Neonatologia, AOUP *and the *Otology and Cochlear Implant Centre *of the University hospital of Pisa. TEOAE and AABR tests are executed with the device *Accuscreen Pro-GN Otometrics*- by an audiometrist with the collaboration of dedicated medical personnel. Tests are performed following the guidelines described above. TEOAE are executed on all newborns. The test is repeated before discharge when the first examination produces a positive result. If the second test still produces a positive (*refer*) result neonates undergo AABR test.

AABR are immediately executed in neonates with audiological risk factors (risk factors are based on those reported on the JCIH Position Statement 2000 and 2007). When AABR produces a positive result further audiological tests are executed. Hearing impairment severity degree is classified according to the BIAP (*Bureau International d'AudioPhonologie*) criteria (mild between 21 and 40 dB of hearing loss; medium between 41 and 70 dB; severe between 71 and 90 dB and profound over 91 dB of hearing loss). If hearing loss (HL) is confirmed further biochemical and instrumental tests are carried out. When the cause of HL can't be detected, DNA is extracted from the Guthrie card which had been stored in the first days from birth to search for CMV genome. DNA is extracted with DNA blood minikit (final eluition 50 uL) and visualized on agarosio gel.

## Results

From the 20th of November 2005 to the 30th of April 2009 8113 neonates born in the Neonatal Unit of Santa Chiara Hospital (Pisa) have undergone newborn hearing screening (Table 1).

**Table 1:** Results of Pisa's experience from the 20th of November 2005 to the 30th of April 2009

	Total number	Neonates with audiological risk factors	Neonates without audiological risk factors
**Screened neonates**	**8.113**	**492 (6.06%)**	**7621(93.9%)**

**Positive screening result at level II test**	**84 (1.04%)**	**39 (46.42%)**	**45 (53.57%)**

**False-positive results**	**44 (0.54%)**	**15 (34.09%)**	**29 (65.9%)**
at first ABR	24 (54.5%)	9	15
at second ABR	15 (34.09%)	4	11
at third ABR	5 (11.36%)	2	3

**No-show patients**	**6**	**2**	**4**

**Hearing impaired patients**	**34 (4.2‰)**	**22**	**12**

**Unilaterally hearing impaired patients**	**8 (0.99‰)**	**1**	**7**

**Bilaterally hearing impaired patients**	**26 (3.2 ‰)**	**21 (80.77%)**	**5 (19.23%)**
mild HL	5 (0.6 ‰)		
Medium HL	11 (1.4 ‰)		
severe/profound HL	10 (1.2 ‰)		

7621 neonates (93.9%) without risk factors executed only the TEOAE test (they executed AABR only if the first test was *refer*). 492 (6.1%) of them had audiological risk factors and thus underwent TEOAE + AABR test. 84 patients (1,04%), among which 39 with risk factors and 45 without risk factors failed both first (TEOAE) and second level (AABR) tests. These 84 infants underwent audiological evaluation at *Otology and Cochlear Implant Centre *of the University hospital of Pisa. Only 6 of them (0.07% of the 8.113 neonates) (two with risk factors and four without) didn't execute further examinations because parents didn't agree to further tests (2 cases); because the hospital was unable to get in touch with the parents (2 cases); because of life-threatening conditions (1 case); or because they died (1 case). 78 infants underwent clinical ABR and further tests. 24 resulted *pass *at the first test. 15 failed the first test but resulted *pass *at the second ABR test. In other 5 patients the third examination with AABR gave a *pass *result. Finally 44 patients resulted false-positives (the 0,54% of the screened newborns).

34 neonates (4,2‰) had a final diagnosis of hearing impairment. 8 patients (0.99‰) (only one had risk factors) had unilateral hearing loss (6 mild, 1 medium and 1 severe). 26 patients (3,2‰) had a final diagnosis of bilateral hearing impairment : 5 mild (0,6‰), 11 medium (1,4‰) and 10 (1,2‰) severe/profound HL. 21 of them (80,77%) had risk factors (9 neonates with severe hearing impairment had risk factors: 7 had suffered from neonatal respiratory distress, 1 had a family history of deafness, 1 had craniofacial anomalies and had suffered from neonatal respiratory distress). In one patient auditory neuropathy was diagnosed.

Among the 26 bilaterally hearing impaired patients: 12 cases were prematurely born or had suffered from respiratory problems (neonatal asphyxia or respiratory distress); in 2 patients connexine 26 gene mutations were found (heterozygous mutation V37I plus heterozygous mutation V95M on GJB2 gene; heterozygous mutation G59A on gene GJB2); 1 patient had family history of infantile deafness; 4 patients had syndromes related to hearing impairment (1 case of Charge syndrome with the sporadic mutation c.5300+1G→T in the *CHD7 *gene; 1 case of Diamond Blackfan Anemia; 1 case of 5q11.2-q13 duplication; 1 case of malformative syndrome in a female with q11 deletion plus partial trisomy of chromosome 6); 3 patients had congenital CMV infections (one of them was positive for CMV and heterozygous mutation R127H on GJB2 gene).

In four neonates hearing loss was classified as idiopathic. DNA was extracted from the Guthrie card of these 4 patients to search for CMV genome but the result was negative.

## Discussion and Conclusions

Universal newborn hearing screening represents the only way to early identify neonates with hearing impairment. If HL is treated precociously with a prosthetic/rehabilitative treatment within few months from birth, the serious consequences that a hearing impairment can have on the language development and on the future communication capabilities of the baby can be minimized or prevented [40-45].

TEOAE is the most adequate test because it is accurate, economic and of simple and rapid execution [22,41,46]. Following the guidelines of JCIH an additional test (AABR) has been introduced for neonates with audiological risk factors [7,22]. AABR is necessary to identify patients with auditory neuropathy but it is also important to test neonates whose TEOAE test was *refer *in order to reduce the number of falsepositives and avoid further examinations.

The 20-30% of infant hearing impairment is represented by progressive or late-onset hearing loss. Newborn screening is not sufficient to identify this kind of hearing impairment [39]. That's the reason why it's necessary to establish an audiological follow-up program, especially in infants at risk.

Our group has formulated, according to the work of the 2007 *JCIH *Position Statement and other international statements, the guidelines for the execution of the audiological screening in Tuscany. The following are the main peculiar contents of our guidelines for newborn hearing screening.

• The risk factors for auditory neuropathy are investigated with special attention

• A screening manager is nominated in each facility

• Tests are executed mainly by audiometrists with the collaboration of dedicated and trained medical personnel

• Even when the screening is *refer *in only one side, neonates undergo further audiological tests

• All hearing impaired newborns are early tested for congenital exposure to CMV infection in order to distinguish it from CMV infections caught later

• An audiological follow-up program is advised in patients with risk factors for progressive or late-onset hearing loss.

In our screening program the percentage of false-positive cases was quite low when compared to literature reports (0.54%). The 34.1% of false positive cases are patients with risk factors.

We also underscore the importance of a follow-up in newborn who have failed the newborn hearing screening. In some cases (24 were *pass *at the first ABR, 15 at the 2nd, 5 at the 3rd) the hearing threshold of ABR improved after the repetition of the test, probably in relation to a maturation of the auditory system. Some patients with hearing threshold lower than 40 dB at the first ABR test gained a normal hearing threshold over a follow-up of six months. Physiological immaturity of central auditory nervous system and physiological narrowness of auditory canal can thus explain most of false positive results if audiological tests are performed in the first few days after birth.

So before giving a final diagnosis we repeat the ABR test 3 times at least in the first six months of age. The incidence of bilateral hearing impairment in our experience (3.2‰) is a little higher than that found in international and national literature.

Perhaps this difference is due to the fact that our data have been collected in a level III facility with a neonatal intensive care unit so many neonates which have been tested have undergone neonatal intensive care for more than 5 days, they have required assisted ventilation, they have been exposed to ototoxic medications or they have gained high levels of hyperbilirubinemia (80.77% of neonates with bilateral HL had audiological risk factors).

It's important to identify the etiology of HL so that the treatment can be more individualized. In our study we could not identify the causes of HL in only 4 patients. In 12 neonates (46.15% of bilaterally hearing impaired patients) the main cause is premature birth or neonatal respiratory distress.

The implementation of this screening program has required a considerable organizational effort and the employment of dedicated personnel which has absorbed resources otherwise available to other audiological activities. Four patients referred to the screening program did not turn up for the tests despite repeated invitations from the hospital. This situation occurred in 4.76% of the referred neonates - 0.05% of all the screened patients. It's also essential, in our opinion, family pediatricians to be involved in a follow-up program in order to detect children with progressive or lateonset HL.

## Competing interests

The authors declare that they have no competing interests.

## Authors' contributions

PG and SB conceived the study, participated in its design and coordination and drafted the manuscript. AL participated in the design of the study and performed audiological screening (TEOAE and AABR). FF participated in the design of the study, performed further audiological investigation and drafted the manuscript. SL participated in the design of the study, performed audiological screening and drafted the manuscript. AB and AB participated in drafting the manuscript and in the analysis of the results.

All authors have read and approved the final manuscript.
